# In Vitro Evaluation of Antidiabetic, Antioxidant, and Anticholinergic Properties of Different Parts of Bilberry ( *Vaccinium myrtillus* L.) From the Eastern Black Sea Region, With LC/MS–MS Analysis of Their Chemical Composition in Food Applications

**DOI:** 10.1002/fsn3.70249

**Published:** 2025-05-02

**Authors:** Hafize Yuca, Beyzanur Ayar, Bilge Aydın, Furkan Çoban, Songül Karakaya

**Affiliations:** ^1^ Department of Pharmacognosy, Faculty of Pharmacy Atatürk University Erzurum Turkey; ^2^ Department of Pharmacognosy, Faculty of Pharmacy Erzincan Binali Yıldırım University Erzincan Turkey; ^3^ Department of Field Crops, Faculty of Agriculture Ataturk University Erzurum Turkey; ^4^ Department of Pharmaceutical Botany, Faculty of Pharmacy Atatürk University Erzurum Turkey

**Keywords:** Alzheimer's disease, antioxidant, bilberry, chlorogenic acid, diabetes, *Vaccinium myrtillus*

## Abstract

Diabetes, the most prevalent metabolic disorder, is closely linked to Alzheimer's disease, with DM patients showing a two‐fold increased risk of developing AD. Bilberry ( *Vaccinium myrtillus* L.) is a prominent wild berry, widely used in juice and food production. This study aimed to evaluate and compare the phytochemical composition and biological activities of different parts of wild *V. myrtillus* plants, including branches, leaves, and fruits. Using LC–MS/MS, the study quantifies total phenolics, flavonoids, and tannins in each part. Biological activities are assessed through antioxidant activity (ABTS and DPPH assays), anticholinesterase (neuroprotective potential), and antidiabetic effects. The experiments revealed that the Leaf of Yer Ligarba (YLL) extract had the highest values in terms of total phenolic content, total flavonoid content, and total tannin content. Among the extracts, the highest radical scavenging effect was observed with YLL at 86.67% against DPPH. The leaf extracts, Leaf of Dal Ligarba (DLL) and YLL, demonstrated the highest scavenging effects, with inhibition rates of 99.37% and 99.37%, respectively. At one of the common concentrations, 100 μg/mL, acarbose showed 13.12% inhibition, while the Branch of Dal Ligarba (DLB) extract exhibited 98.67% *α*‐glucosidase enzyme inhibition. The highest acetylcholinesterase and butyrylcholinesterase enzyme inhibition activities were observed in the Branch of Yer Ligarba (YLB; 27.08%) and the Fruit of Dal Ligarba (DLF; 16%), respectively. Chlorogenic acid was the most abundant phenolic compound, particularly in the DLL 58,569.80 ng/mL. This study highlighted the significant phytochemical content and biological activities of wild bilberry extracts, demonstrating their potential for managing diabetes and neurodegenerative diseases, particularly Alzheimer's.

## Graphical abstract

This study evaluates the phytochemical composition and biological activities of wild bilberry ( *Vaccinium myrtillus* L.) extracts. Leaf extracts showed strong antioxidant, antidiabetic, and anticholinesterase effects, highlighting their potential in managing diabetes and Alzheimer's disease.

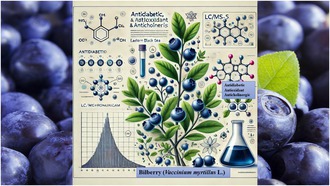

## 1 Introduction

It highlights the inherent connection between food and medicine, forming the basis for dietary therapy and nutrition‐based health management. As this concept gains global recognition and continues to develop, there is a clear transition from the passive use of “medicinal‐like food supplements” toward a more deliberate and informed approach to “therapeutic nutrition.” (Sun‐Waterhouse et al. 2024).

Diabetes mellitus (DM) is a widespread metabolic disorder characterized by chronic hyperglycemia. Currently affecting around 642 million people, this number is expected to rise to 800 million by 2045. In 2016, DM caused 1.6 million deaths, with approximately 25% of patients developing diabetic foot ulcers—leading to infections, prolonged hospital stays, amputations, and reduced quality of life (Haile et al. 2025). Chronic hyperglycemia in diabetic patients can lead to various complications, including nephropathy, cardiovascular disorders, neuropathy, as well as ocular and hepatic impairments (Zheng et al. 2024). DM continues to pose a significant global health challenge, marked by rising prevalence and increasing mortality rates (Ghazaly et al. 2025).

Alzheimer's disease (AD) is a progressive neurodegenerative disorder characterized by cognitive decline. With no known cure or prevention, it is projected to become the second leading cause of death within two decades (Cuní‐López et al. 2024).

AD is a multifactorial neurodegenerative disorder, with metabolic dysfunction—especially DM—being a key contributor. DM, the most common metabolic disease, doubles the risk of developing AD. Even prediabetes is associated with cognitive decline and hippocampal atrophy. Insulin resistance, central to DM, is also involved in AD pathogenesis, leading to the term “type 3 diabetes.” Oxidative stress (OxS) plays a major role in both type 2 diabetes (T2D) and AD, with amyloid‐β and tau pathologies linked to OxS and disrupted glucose metabolism. Insulin/IGF signaling impairments, hyperglycemia, and resulting neuroinflammation further connect DM to neurodegeneration. T2D and AD share overlapping genetic, aging‐related, and pathological mechanisms, including amyloid accumulation (Arnold et al. 2018; Ha et al. 2020; Janson et al. 2004; Rosales‐Corral et al. 2015).

Most *Vaccinium* L. species originate from North America, South America, and East Asia (Çolak 2013). Bilberry ( *V. myrtillus* L.) is a well‐known wild berry native to Northern Europe, commonly used in food and beverage production. This low‐growing shrub yields dark purple berries rich in anthocyanins, comprising about 0.5% of the fresh fruit. Bilberries are consumed fresh or in various processed forms such as frozen, dried, juices, jams, supplements, and more recently, fermented products. Numerous studies have confirmed the health‐promoting properties of *Vaccinium* species, including antioxidant, antimicrobial, anti‐inflammatory, and protective effects against diabetes, obesity, cancer, cardiovascular, and neurodegenerative disorders. As their commercial use expands, so does the generation of by‐products like leaves, which are rich in bioactive phenolics. These include anthocyanins (e.g., cyanidin, malvidin), flavonoids (e.g., quercetin, isoquercetin), phenolic acids (e.g., gallic, syringic, caffeic), and iridoids—compounds with both health and economic value (Kopystecka et al. 2023; Martău et al. 2023). In Turkey, *V. myrtillus* naturally grows at high altitudes in regions such as Kazdağ, Uludağ, Ugazdağ, and the Eastern Black Sea. In the Black Sea region, it thrives in acidic soils and harsh conditions, often coexisting with spruce, beech, rhododendron, alder, pine, and ferns. The plant spreads via rhizomes, forming dense ground cover near forested areas. Commonly known as “Çobanüzümü” in Turkish, it is also referred to locally by various names, including “likapa,” “ligarba,” “ayı üzümü,” “lifos,” “lifora,” “çalı çileği,” “yaban mersini,” “yer ligarbası,” and “dal ligarbası.” (Bozdağ 2019; Patan 2017). It is a deciduous, rhizomatous perennial shrub with above‐ground shoots ranging from 10 to 60 cm in height. The leaves are 1–3 cm long, entirely glabrous, shiny, with net‐like veins on the underside, and serrulate, toothed margins. It flowers between May and June, with blossoms occurring singly or in pairs in the leaf axils. The fruits are round, bluish with a frosted appearance, and the flesh of the fruit is also pigmented (Bozdağ 2019; Patan 2017; Ritchie 1956). Bilberries, valued as a natural food and beverage source, are rich in nutrients and bioactive compounds. They are widely consumed as dietary supplements and pharmaceutical products due to their health benefits. *V. myrtillus* berries have been popular globally since ancient times, traditionally used to address ailments like cough, fever, kidney stones, and digestive issues. They are also applied topically for conditions such as diarrhea, vomiting, hemorrhoids, skin ulcers, and to enhance visual function. Additionally, these berries play a significant role in traditional diets and are incorporated into various medicines. Wild blueberries are available fresh, frozen, dried, or processed into jams, juices, and concentrated supplements. Infusions made from *V. myrtillus* leaves, known as “antidiabetic teas,” have been traditionally used for their hypoglycemic properties (Asgary et al. 2016; Bujor et al. 2016; Cignarella et al. 1996; Gaspar et al. 2021; Pires et al. 2020; Tadić et al. 2021). *V. myrtillus* is a medicinal plant from the Ericaceae family, traditionally used for its rich phytochemical content. Its anthocyanin‐rich berries support eye and vascular health, while the leaves contain compounds like quercetin, chlorogenic acid, and proanthocyanidins with antidiabetic and antioxidant effects. The plant offers broad pharmacological benefits, including anti‐inflammatory, antimicrobial, and cardioprotective properties (Chehri et al. 2022; Sharma and Lee 2022). A recent review examined the phytochemical content and health effects of bilberry fruits and leaves. While traditionally promoted for managing type 2 diabetes, vision problems, and circulatory disorders, the evidence remains inconsistent. Many claims, especially for diabetes and vision, stem from outdated or unverified sources. The most promising results were observed in studies on dyslipidemia and inflammation, though more robust clinical trials are still needed (Vaneková and Rollinger 2022). Bilberry leaves are generally considered non‐toxic; however, their high tannin content warrants caution. Tannins may cause constipation and interfere with nutrient absorption by forming complexes with proteins and digestive enzymes. Prolonged use can impair iron absorption, potentially leading to iron deficiency, particularly in vulnerable groups such as children and the elderly (Ștefănescu et al. 2022).

The primary objective of this study is to comprehensively evaluate and compare the phytochemical composition and biological activities of various parts of *V. myrtillus* plants, collected from the wild. The analysis encompasses the branches, leaves, and fruits, with a detailed investigation of their phytochemical profiles using LC–MS/MS. Specifically, the study focuses on quantifying the total phenolic content, total flavonoid content, and total tannin levels in each plant part. Additionally, their biological potential is assessed through antioxidant activity using ABTS and DPPH radical scavenging assays, anticholinesterase activity as an indicator of neuroprotective potential, and antidiabetic activity to evaluate their efficacy in diabetes management. This comprehensive analysis aims to provide valuable insights into the functional and medicinal properties of different parts of *V. myrtillus* , contributing to its potential applications in healthcare and nutrition.

## 2 Material and Method

### 2.1 Plant Material

**FIGURE 1:**
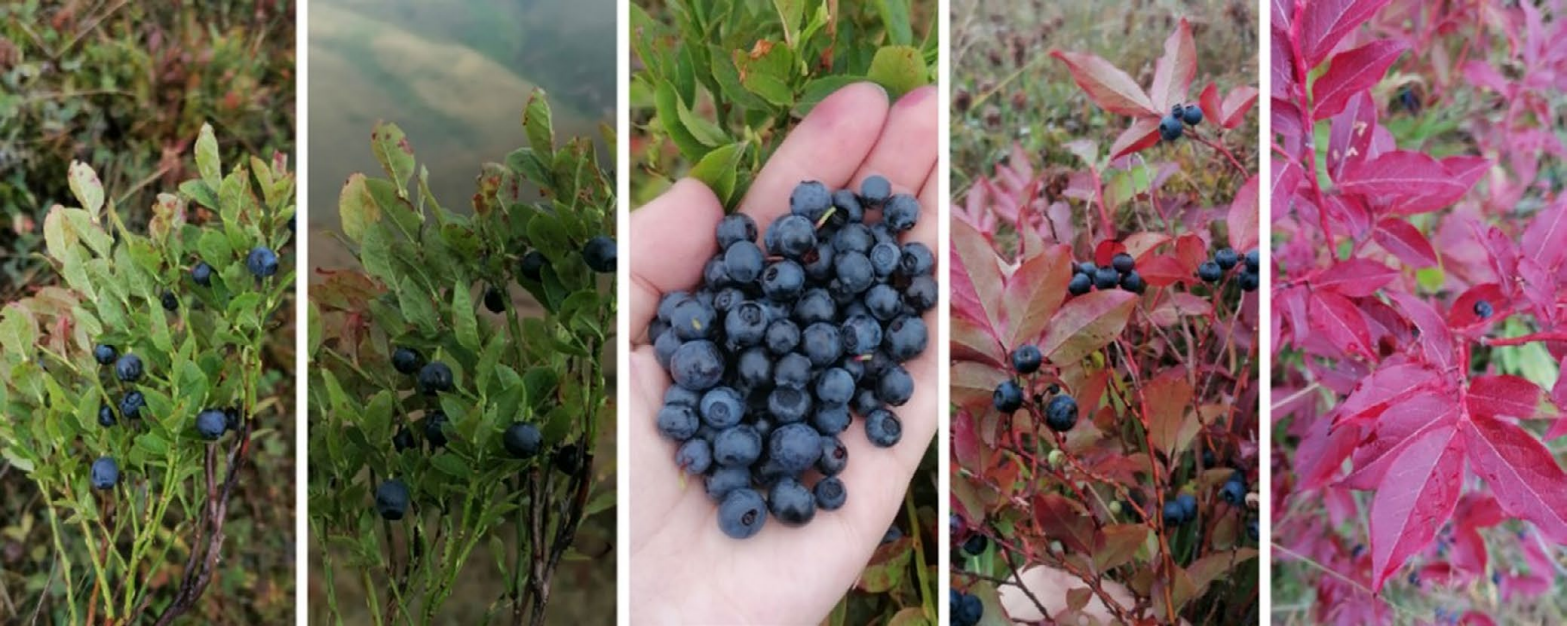
Branches, leaves, and fruits of the *V. myrtillus* (by Beyzanur Ayar).

The plant materials used in this study were collected from Kutlusu Yaylası, located in the Köprübaşı district of Trabzon, in September 2023. The different parts of two distinct plants were assigned specific codes for identification. Details regarding the plant parts, their names, and the collection location and time are provided in Table 1. The specimens collected from the wild are referred to as “dal ligarba” and “yer ligarba,” a distinction made based on the variations in fruit size between the two plants. Herbarium specimens, labeled AUEF 1413, 1414, 1415, and 1416, are carefully preserved at the Biodiversity Application and Research Center of Atatürk University. Photos of branches, leaves, and fruits of the *V. myrtillus* are presented in Figure 1.

**TABLE 1:** Plant materials used in the study, parts used, place and time of collection.

Plant name	Used part		Collection location	Toplanma Zaman collection date
	Dal Ligarba	Yer Ligarba		
*Vaccinium myrtillus*	Branch (DLB)	Branch (YLB)	Köprübaşı/Trabzon	08/09/2023
	Leaf (DLL)	Leaf (YLL)		
	Fruit (DLF)	Fruit (YLF)		

### 2.2 Chemicals and Equipment Used

#### 2.2.1 Solid Chemicals

Gallic acid monohydrate (Purity ≥ 99%; Isolab, Germany), Sodium carbonate (Na_2_CO_3_; Purity 99.5%–100.5%; Sigma‐Aldrich, Germany), *α*‐Tocopherol (Purity ≥ 96%; Sigma‐Aldrich, Germany), Trolox (Purity 97%; Sigma‐Aldrich, Switzerland), 2,2‐Diphenyl‐1‐picrylhydrazyl (DPPH), 2,2′‐Azino‐bis(3‐ethylbenzothiazoline‐6‐sulfonic acid) (ABTS; Purity ≥ 98%; Sigma‐Aldrich, USA), Potassium persulfate (Purity ≥ 99%; Sigma‐Aldrich, Germany), Rutin (Purity ≥ 94%; Sigma‐Aldrich, China), Aluminum chloride (AlCl_3_; Sigma‐Aldrich, Germany), *α*‐Glucosidase enzyme (derived from *Saccharomyces cerevisiae* ; Sigma‐Aldrich, Israel), and p‐Nitrophenyl‐*α*‐D‐glucopyranoside (Purity ≥ 99%; Sigma‐Aldrich, Germany).

#### 2.2.2 Solvents

Methanol (Honeywell Riedel‐de‐Haën, Germany; Purity ≥ 99.8%), Dimethyl sulfoxide (DMSO, Germany; Purity ≥ 99.9%), Distilled water, Folin–Ciocalteu Reagent (FCR; Sigma‐Aldrich, Germany), Ethanol (Sigma‐Aldrich, Germany, Purity ≥ 99.8%), Phosphate buffer (pH = 7.4, 0.1 M), Diluted HCl (Hydrochloric acid), and Potassium phosphate buffer (pH = 6.9, 0.1 M).

#### 2.2.3 Equipment

Oven: Memmert Typ. UM 500, Ultrasonic Bath: Bandelin Sonorex RK 255 H, Analytical Balance: Precisa XB 620 M, Spectrophotometer: Thermo Scientific Multiscan Go, Glass Mortar, Shaking Water Bath: Miprolab Mcs 45 Shaking Water Bath.

### 2.3 Extraction

The plant parts, which were separated, dried in the open air and shade, and crushed, were placed in a glass mortar and ground thoroughly with a pestle. Different amounts of each plant part were precisely weighed using an analytical balance and transferred into individual round‐bottom flasks. To each flask, 90 mL of methanol was added, and the mixtures were macerated overnight at room temperature. Although the plant material quantities varied, a fixed solvent volume was used to allow comparison of extraction yields under consistent conditions. The following day, the mixtures were extracted for 1 day at 40°C and 100 rpm in a shaking water bath. After extraction, the materials were removed from the shaking water bath and filtered through a common funnel into beakers and small jars, removing the plant residue from the solution. The obtained filtrate was evaporated to dryness on a rotary evaporator.

### 2.4 Total Phenolic Compound Quantification

The total phenolic content of *V. myrtillus* extracts was determined using a method developed by Folin and Denis and modified by Singleton and our team, with the results expressed as gallic acid equivalents (Folin and Denis 1912; Slinkard and Singleton 1977). A 1 mg/mL stock solution of gallic acid was prepared as the standard compound. From this stock, a series of sub‐concentrations (100, 200, 300, 400, 500, and 600 μg/mL) were prepared. To these solutions, Folin–Ciocalteu reagent (FCR) and Na_2_CO_3_ were added according to the experimental procedure. The standard curve for gallic acid was obtained by recording the absorbance values at 760 nm against distilled water as a blank. Using the equation obtained from the standard curve (*r* ^2^ value 0.99), the total phenolic content of the extracts was calculated in terms of gallic acid equivalents according to their absorbance at a 1 mg/mL concentration. Each analysis was performed in triplicate.

### 2.5 Total Flavonoid Content Determination

The total flavonoid content of the extracts was determined based on the method described by Liu et al. (2007) with slight modifications, using rutin as the standard compound. The rutin standard compound, which contains a flavonoid structure, was dissolved in DMSO at a concentration of 1 mg/mL. A series of dilutions were prepared from the stock solution. These sub‐concentrations were treated with Folin–Ciocalteu reagent (FCR) and Na_2_CO_3_, following the method of Nikolaeva et al. (2022). According to the experimental procedure, absorbance values were recorded against the concentration, and the rutin standard curve was generated (*r* ^2^ value 0.99). The total flavonoid content of the extracts was calculated as a percentage of rutin and expressed as μg RE/mg extract. Each analysis was conducted in triplicate.

### 2.6 Total Tannin Content Determination

The total tannin content of the extracts obtained from different parts of *V. myrtillus* was determined using the modified Folin–Ciocalteu method, as described by Makkar (2003). Tannic acid was used as the standard compound, and solutions were prepared in the concentration range of 100–600 μg/mL. To prepare the samples for the extracts, a stock solution of each extract was prepared at a concentration of 1 mg/mL. Both the standard solutions and the extracts were treated with 0.5 mL of Folin–Ciocalteu reagent (FCR). Following the procedure, 1.5 mL of 2% sodium carbonate (Na_2_CO_3_) solution was added. After the incubation period, the absorbance of the samples was read at 725 nm against a blank prepared with distilled water. The tannic acid equivalents (TAE) corresponding to the absorbance values of the samples were determined using the equation obtained from the standard curve (*r* ^2^ value 0.99). The results are presented as tannic acid equivalents (TAE) in micrograms. Each analysis was conducted in triplicate.

### 2.7 Antioxidant Capacity Assays

#### 2.7.1 DPPH Radical Scavenging Capacity Determination

The DPPH^•^ scavenging capacity was determined based on the method developed by Blois (1958) with slight modifications (Aydın et al. 2023). In this method, *α*‐tocopherol and trolox were used as standards, and a 1 mM DPPH^•^ solution was employed to test antioxidant activity. The percentage inhibition values of the standards at various concentrations (1–100 μg/mL) against DPPH^•^ were examined, and the concentration range for the samples to be tested was determined. The calibration curve, obtained by taking into account the percentage inhibition values of the standard substances at the selected concentrations, was used to determine the concentration range of the samples to be studied (*r* ^2^ value 0.99). A series of dilutions of the extracts within the concentration range of 10–100 μg/mL were prepared, and their antioxidant activities were measured against the 1 mM DPPH^•^ solution. All measurements were recorded at 517 nm against a blank consisting of 99% ethanol. Each analysis was performed in triplicate. The DPPH^•^ scavenging capacity of the extracts was calculated using the following equation:

$$\text{Inhibition}\%=\left[\left(A_{\text{control}}-A_{\text{sample}}\right)/A_{\text{control}}\right]\times 100$$

#### 2.7.2 ABTS Cation Radical Scavenging Capacity Determination

The ABTS^•+^ scavenging capacities of *V. myrtillus* extracts were determined based on the method developed by Re et al. (1999) with slight modifications (Aydın et al. 2023). In this method, α‐tocopherol and trolox were used as standards, and a 2 mM ABTS^•+^ solution was employed to test antioxidant activity. The percentage inhibition values of the standards at various concentrations (1–100 μg/mL) against ABTS^•+^ were examined, and the concentration range for the samples to be tested was decided. The calibration curve was prepared as in the DPPH^•^ test, and the concentrations to be studied were determined (*r* ^2^ value 0.99). A series of dilutions of the extracts within the concentration range of 10–100 μg/mL were prepared, and their antioxidant activities were measured against the 2 mM ABTS^•+^ solution. All measurements were recorded at 734 nm against a blank consisting of phosphate buffer. Each analysis was performed in triplicate. The ABTS^•+^ scavenging capacity of the extracts was calculated using the following equation:

$$\text{Inhibition}\%=\left[\left(A_{\text{control}}-A_{\text{sample}}\right)/A_{\text{control}}\right]\times 100.$$

### 2.8 Antidiabetic Activity Determination

#### 2.8.1 *α*‐Glucosidase Enzyme Inhibition Activity Determination

The *α*‐glucosidase inhibitory activity was determined using a method developed by Bachhawat et al. (2011), with some modifications. In a 96‐well microplate, 50 μL of phosphate buffer (50 mM, pH 6.9), 10 μL of α‐glucosidase enzyme (1 Unit/mL), and 20 μL of plant extracts (with concentrations ranging from 1 to 5000 μg/mL) were mixed and incubated at 37°C for 5 min. Then, 20 μL of 3 mM p‐nitrophenyl‐α‐D‐glucopyranoside (pNPG) was added as a substrate, and the mixture was incubated at 37°C for 30 min. The reaction was completed by adding 50 μL of 0.1 M sodium carbonate. All solutions were prepared in a buffer system. Acarbose was used as the positive control. The amount of yellow p‐nitrophenol (pNP) formed was measured at 405 nm. Each determination was repeated three times. The results were calculated using the following equation:

$$\text{Inhibition}\%=\left[\left(A_{\text{control}}-A_{\text{sample}}\right)/A_{\text{control}}\right]\times 100$$

#### 2.8.2 *α*‐Amylase Enzyme Inhibition Activity Determination

The *α*‐amylase inhibitory activity was determined according to a method developed by Nampoothiri et al. (2011). In equal volumes (100 μL), the sample (with concentrations ranging from 1 to 5000 μg/mL) and a 1% starch solution were incubated in 20 mM sodium phosphate buffer (pH 6.9, with 6 mM sodium chloride) at 25°C for 10 min in a microplate. Then, 100 μL of porcine pancreatic α‐amylase (0.5 mg/mL) was added to each well, and the samples were incubated at 25°C for an additional 10 min. The reaction was stopped by adding 200 μL of dinitrosalicylic acid (DNS) color reagent, and the tubes were incubated at 100°C for 5 min. The samples were cooled to room temperature, and 50 μL from each tube was transferred to a 96‐well microplate. 200 μL of distilled water was added to dilute the reaction mixture in each well, and absorbance was measured at 540 nm. The results were compared with the control. Acarbose was used as the positive control. The results were calculated using the following equation. Each determination was repeated three times.

$$\text{Inhibition}\%=\left[\left(A_{\text{control}}-A_{\text{sample}}\right)/A_{\text{control}}\right]\times 100$$

### 2.9 Acetylcholinesterase (AChE) and Butyrylcholinesterase (BChE) Enzyme Inhibition Activity Assays

The acetylcholinesterase (AChE) and butyrylcholinesterase (BChE) enzyme activity assays were performed with slight modifications based on the method developed by Ingkaninan et al. (2000). 125 μL of 5,5′‐dithiobis‐(2‐nitrobenzoic acid) (3 mM, DTNB, Ellman's Reagent), 25 μL of substrate (15 mM, acetylthiocholine iodide for AChE and butyrylthiocholine iodide for BChE), 50 μL of Tris–HCl buffer (50 mM, pH: 8), and 25 μL of sample were mixed in a 96‐well plate. Finally, 25 μL of enzyme (AChE or BChE) was added to the mixture, and for the AChE enzyme inhibition assay, the reaction was incubated for 10 min, while for the BChE enzyme inhibition assay, it was incubated for 15 min. The absorbance was then measured spectrophotometrically at 405 nm. Donepezil was used as the positive control. Each assay was performed in triplicate. The percentage of inhibition was calculated using the following equation:

$$\text{Inhibition}\%=\left[\left(A_{\text{control}}-A_{\text{sample}}\right)/A_{\text{control}}\right]\times 100$$

### 2.10 Quantification by LC–MS/MS Method

The qualitative and quantitative analysis of secondary metabolites in the samples will be performed using the Agilent 6460 Triple Quadrupole Liquid Chromatography–Tandem Mass Spectrometry (LC–MS/MS) system, developed by combining chromatography and spectroscopy systems at the Atatürk University Eastern Anatolia High Technology Application and Research Center (DAYTAM). For the separation of the analytes, an Agilent 1260 HPLC system equipped with an Agilent Poroshell 120 EC‐C18 (4.6 × 100 mm, 3.5 μm) column will be used. The HPLC system operates in positive ion mode with electrospray ionization (ESI). Initially, the protonated product ion [M + H]^+^ for each compound will be determined using a standard scanning mode for detection and quantification, with the standard solutions prepared at specific concentrations for each compound. The chromatography process will be carried out using a gradient elution technique. The standard compounds will be dissolved in methanol at a concentration of 100 μg/mL and then diluted with methanol to create a calibration curve. The gradient profile will be set as follows: from 0 to 4 min, 5% B; from 4 to 7 min, 20% B; from 7 to 14 min, 90% B; at 15 min, 90% B; from 15 to 15.1 min, 5% B; and from 15.1 to 17 min, 5% B. The flow rate will be set at 0.4 mL/min. A small volume (5 μL) of the sample will be injected, and the total analysis time will be 17 min. The mass spectrometry conditions will be set as follows: nitrogen gas temperature of 350°C, a flow rate of 12 L/min, carrier gas temperature of 250°C, a flow rate of 5 L/min, and nebulizer gas pressure of 55 psi. Data collection and analysis will be performed using the Agilent MassHunter Workstation, which includes a full mass scan within the 50–1300 m/z range. For the LC–MS/MS method, standard solutions of 1 mg/μL concentration will be prepared from each extract and material using HPLC‐grade methanol (Yuca et al. 2023).

### 2.11 Statistical Analysis

All assays were performed in triplicate. To assess statistical significance, the Kruskal–Wallis test was employed.

### 2.12 Data Analysis

Pearson correlation analysis was performed to determine the relationships among the variables. All analyses were conducted using R software (RStudio 2024.12.0). Correlation coefficients and significance levels were calculated using the psych and PerformanceAnalytics packages. Correlation matrices were visualized with the help of the corrplot and GGally packages. Significance levels were considered as *p* < 0.05, *p* < 0.01, and *p* < 0.001. To reveal the underlying patterns among variables, principal component analysis (PCA) was applied. PCA was performed using the FactoMineR and factoextra packages, and the results were visualized with a biplot illustrating the distribution of samples and the loadings of variables.

## 3 Results

### 3.1 Extraction

The highest yield percentage was observed for the YLF, which reached 30.26%, while the lowest yield was recorded for the DLF, at 9.65%. This variation in yield percentages highlights the differences in extraction efficiency among the different plant parts, with YLF demonstrating the most efficient extraction process and DLF yielding the least amount of extract. The amount of powdered material and the percentage yields of extracts from different parts of *V. myrtillus* are presented in Table 2.

**TABLE 2:** Amount of powdered material, percentage yields of extracts from different parts of *V. myrtillus* .

Samples	Powdered material (g)	Extract amount (g)	Yield (%)
DLB	16.66	2.08	12.48
DLL	30.36	6.08	20.02
DLF	22.84	2.20	9.65
YLB	18.46	2.17	11.75
YLL	16.36	2.35	14.37
YLF	25.57	7.74	30.26

Abbreviations: DLB, Branch of Dal Ligarba; DLF, Fruit of Dal Ligarba; DLL, Leaf of Dal Ligarba; YLB, Branch of Yer Ligarba; YLF, Fruit of Yer Ligarba; YLL, Leaf of Yer Ligarba.

### 3.2 Total Phenolic Compound Quantification

The total phenolic content of the extracts was calculated in terms of GAE (Gallic Acid Equivalent) using the formula obtained from the graph of absorbance versus concentration recorded for gallic acid (Table 3).

**TABLE 3:** The total phenolic content of *V. myrtillus* extracts.

Samples	Total phenolic content (μg GAE/mg extract ± SD)
DLL	120.81 ± 0.0025
YLL	142.22 ± 0.0052
DLB	131.98 ± 0.0016
YLB	132.62 ± 0.0041
DLF	22.41 ± 0.0008
YLF	17.17 ± 0.0044

Abbreviations: DLB, Branch of Dal Ligarba; DLF, Fruit of Dal Ligarba; DLL, Leaf of Dal Ligarba; YLB, Branch of Yer Ligarba; YLF, Fruit of Yer Ligarba; YLL, Leaf of Yer Ligarba.

The extract with the highest total phenolic content was identified as YLL, with a value of 142.24 μg GAE/mg extract. The branches and leaves of the plants exhibited similar values, with YLB at 132.62 μg GAE/mg extract, DLB at 131.98 μg GAE/mg extract, and DLL at 120.81 μg GAE/mg extract, following closely behind. In contrast, the fruit extracts showed lower gallic acid equivalent values, with DLF at 22.41 μg GAE/mg extract and YLF at 17.17 μg GAE/mg extract.

### 3.3 Total Flavonoid Content Determination

The flavonoid content of *V. myrtillus* extracts, expressed as rutin equivalents, was determined using the rutin standard graph. The results were calculated as percentages and are presented in μg RE/mg extract in Table 4.

**TABLE 4:** The total flavonoid content of *V. myrtillus* extracts.

Samples	Total flavonoid content (μg RE/mg extract ± SD)
DLL	1454.33 ± 0.0025
YLL	1754.33 ± 0.0052
DLB	1610.67 ± 0.0016
YLB	1619.67 ± 0.0041
DLF	76.67 ± 0.0008
YLF	3.33 ± 0.0044

Abbreviations: DLB, Branch of Dal Ligarba; DLF, Fruit of Dal Ligarba; DLL, Leaf of Dal Ligarba; YLB, Branch of Yer Ligarba; YLF, Fruit of Yer Ligarba; YLL, Leaf of Yer Ligarba.

When comparing the total flavonoid contents, it was determined that the YLL extract had the highest rutin equivalent content, with a value of 1754.33 μg RE/mg extract. Following this, DLL showed a value of 1454.33 μg RE/mg extract, and the YLB and DLB extracts showed very close values of 1619.67 and 1610.67 μg RE/mg extract, respectively. For the fruit extracts, DLF and YLF, the values were calculated as 76.67 and 3.33 μg RE/mg extract.

### 3.4 Total Tannin Content Determination

Total tannin content determination results for the extracts were carried out using the tannic acid standard graph. The results were calculated as percentages and are presented in μg TAE/mg extract in Table 5.

**TABLE 5:** The total tannin content of *V. myrtillus* extracts.

Samples	Total tannin content (μg TAE/mg extract ± SD)
DLL	226.39 ± 0.0025
YLL	276.39 ± 0.0052
DLB	252.44 ± 0.0016
YLB	253.94 ± 0.0041
DLF	ND
YLF	ND

Abbreviations: DLB, Branch of Dal Ligarba; DLF, Fruit of Dal Ligarba; DLL, Leaf of Dal Ligarba; YLB, Branch of Yer Ligarba; YLF, Fruit of Yer Ligarba; YLL, Leaf of Yer Ligarba.

The extract with the highest tannic acid equivalent in total tannin content was determined to be YLL, with a value of 276.39 μg TAE/mg extract. The second highest was the YLB extract, with a value of 253.94 μg TAE/mg extract, followed by DLB with 252.44 μg TAE/mg extract and DLL with 226.39 μg TAE/mg extract. No tannic acid equivalent content was detected in the fruit extracts of the plants.

### 3.5 Antioxidant Capacity Assays

#### 3.5.1 DPPH Radical Scavenging Capacity Determination

Trolox and *α*‐tocopherol were used as standards. Inhibition values were calculated as percentages by recording the absorbance values of standard compounds at varying concentrations and plotting the resulting graph. Based on the maximum percentage inhibition values of the standard compounds, the concentration range for the extracts was determined to be 10–100 μg/mL. The DPPH radical scavenging capacities of the extracts and standard antioxidant compounds at a concentration of 100 μg/mL are provided as % inhibition in Table 6. In the DPPH^•^ scavenging capacity determination, *α*‐tocopherol showed 90.23% inhibition at a concentration of 100 μg/mL, while trolox showed 93.01%. Among the extracts, the highest inhibition was observed with YLL at 86.67% and YLB at 84.39%. The inhibition values for the branch extracts were 80.22% for the branch extract, 77.09% for the leaf extract, and 21.57% for the fruit extract. The lowest inhibition percentage was found for the fruit of the ground plant, which was calculated as 19.97%. Similar to the DPPH^•^ scavenging capacity, the branch and ground plant leaves and branches showed ABTS radical scavenging capacity values very close to *α*‐tocopherol and trolox. The fruits also exhibited lower effects compared to the standard compounds.

**TABLE 6:** Comparison of DPPH^•^ scavenging capacities of samples at 100 μg/mL concentration (% inhibition ± SD).

Samples	DPPH^•^ scavenging capacities (100 μg/mL inhibition % ± SD)
*α‐*Tocopherol	90.23 ± 0.0045
Trolox	93.01 ± 0.0015
DLL	77. 09 ± 0.0196
YLL	86.67 ± 0.0091
DLB	80.22 ± 0.0188
YLB	84.39 ± 0.0124
DLF	21.57 ± 0.0358
YLF	19.97 ± 0.0259

Abbreviations: DLB, Branch of Dal Ligarba; DLF, Fruit of Dal Ligarba; DLL, Leaf of Dal Ligarba; YLB, Branch of Yer Ligarba; YLF, Fruit of Yer Ligarba; YLL, Leaf of Yer Ligarba.

#### 3.5.2 ABTS Cation Radical Scavenging Capacity Determination

Considering the maximum percentage inhibition values obtained from the standard compounds, the concentration range for the extracts to be studied was determined to be 10–100 μg/mL. The ABTS cation radical scavenging capacities of the extracts and standard antioxidant compounds at a concentration of 70 μg/mL are presented as percentage inhibition in Table 7.

**TABLE 7:** Comparison of the ABTS^•+^ scavenging capacities of the samples at a concentration of 70 μg/mL (% inhibition ± SD).

Samples	ABTS^•+^ scavenging capacities (70 μg/mL inhibition % ± SD)
*α‐*Tocopherol	19.57 ± 0.0165
Trolox	99.53 ± 0.0048
DLL	99.37 ± 0.0019
YLL	99.37 ± 0.0015
DLB	99.33 ± 0.0009
YLB	99.35 ± 0.0005
DLF	10.95 ± 0.0236
YLF	11.78 ± 0.0213

Abbreviations: DLB, Branch of Dal Ligarba; DLF, Fruit of Dal Ligarba; DLL, Leaf of Dal Ligarba; YLB, Branch of Yer Ligarba; YLF, Fruit of Yer Ligarba; YLL, Leaf of Yer Ligarba.

In the ABTS scavenging capacity assay, at a concentration of 70 μg/mL, *α*‐tocopherol showed an inhibition of 19.57%, while trolox exhibited an inhibition of 99.53%. The leaf extracts, DLL and YLL, demonstrated the highest scavenging effects with inhibition rates of 99.37% and 99.37%, respectively. The inhibition values for the branch extracts were calculated as 99.35% for YLB and 99.33% for DLB. The lowest scavenging effect was observed in the fruit extracts, with YLF showing an inhibition of 11.78% and DLF showing 10.95%. Based on these data, it was determined that both the local and branch varieties of the plant were significantly more effective than *α*‐tocopherol at the same concentration, showing nearly the same scavenging effect as trolox. The fruit extracts of the local and branch varieties, however, showed inhibition effects similar to *α*‐tocopherol but were less effective than trolox.

In conclusion, the results of the total phenolic, flavonoid, and tannin content assays were found to be consistent with the ABTS and DPPH radical scavenging capacity assay results. In this regard, the highest activity was observed in the branches and leaves of the plants, while the fruit parts exhibited lower activity.

### 3.6 Antidiabetic Activity Determination

In order to determine the antidiabetic activities of *V. myrtillus* extracts, inhibitor activity assays were performed for α‐glucosidase and α‐amylase enzymes and compared with the standard compound acarbose. For the α‐glucosidase assay, a stock solution of 5 mg/mL concentration was first prepared from the plant extracts, and further dilutions were made to obtain seven different concentrations. After observing effective results, the concentrations were adjusted, and further detailed assays were conducted with five of the six extracts at a lower concentration of 8.

At one of the common concentrations, 100 μg/mL, acarbose showed 13.12% inhibition, while the DLB extract exhibited 98.67% inhibition. This activity was followed by YLB with 97.58%, YLL with 82.59%, and DLL with 42.82%. In the fruit extracts, inhibitory activity was found to be 6.53% for DLF and 2.74% for YLF. Based on these findings, it was concluded that the most effective parts of the plant in terms of antidiabetic activity, specifically α‐glucosidase enzyme inhibition, were the branches, followed by the leaves and the fruits. Additionally, it was observed that at the same concentration, the branches and leaves were more effective than acarbose, while the fruits showed lower activity compared to acarbose (Table 8).

**TABLE 8:** α‐Glucosidase and α‐amylase inhibition activity determination results.

Samples	α‐glucosidase inhibition capacity IC_50_ (μg/mL)	α‐amylase inhibition capacity (5000 μg/mL % Inhibition ± SD)
DLL	138.93	ND
YLL	65.98	28.77 ± 4.26
DLB	53.73	ND
YLB	44.60	ND
DLF	1130.98	ND
YLF	566.37	ND
Acarbose	3169.91	65.66 ± 1.41

Abbreviations: DLB, Branch of Dal Ligarba; DLF, Fruit of Dal Ligarba; DLL, Leaf of Dal Ligarba; YLB, Branch of Yer Ligarba; YLF, Fruit of Yer Ligarba; YLL, Leaf of Yer Ligarba.

In another assay for antidiabetic activity, the α‐amylase inhibitor assay was conducted at a single concentration of 5 mg/mL. Acarbose showed 65.66% inhibition, while only YLL exhibited an inhibitory effect of 28.77%. The other parts of the plant were found to be ineffective against the enzyme. In terms of antidiabetic activity, the effect of acarbose on the α‐amylase enzyme was approximately 2.3 times greater than that of the YLL extract.

### 3.7 Acetylcholinesterase (AChE) and Butyrylcholinesterase (BChE) Enzyme Inhibition Activity Assays

In the acetylcholinesterase and butyrylcholinesterase enzyme inhibition assays conducted to evaluate anti‐Alzheimer effects, donepezil was used as the standard compound. The assays were performed at two concentrations: 5 and 100 μg/mL for acetylcholinesterase, and 500 and 1000 μg/mL for butyrylcholinesterase.

In the acetylcholinesterase enzyme inhibition assay, donepezil exhibited effects of 95.68% and 101.34% at concentrations of 5 and 100 μg/mL, respectively. Although no extract showed activity close to that of donepezil, the highest activity was observed in YLB, which showed inhibition of 9.13% and 27.08% at the same concentrations. This activity was followed by YLL, YLF, and DLL. The lowest activities were observed in DLB and DLF extracts (Table 9).

**TABLE 9:** Acetylcholinesterase and butyrylcholinesterase enzymes inhibition activity assay results.

Samples	Acetylcholinesterase		Butyrylcholinesterase	
	5 μg/mL	100 μg/mL	500 μg/mL	1000 μg/mL
DLL	5.15 ± 2.80	16.43 ± 3.54	1.75 ± 1.56	9.58 ± 1.12
YLL	5.62 ± 4.70	25.43 ± 0.82	12.23 ± 1.66	3.87 ± 1.14
DLB	4.52 ± 3.38	22.18 ± 9.62	4.59 ± 0.54	5.17 ± 0.89
YLB	9.13 ± 4.04	27.08 ± 2.61	5.52 ± 1.17	10.56 ± 0.25
DLF	3.25 ± 1.80	15.59 ± 1.53	11.91 ± 2.99	16.37 ± 1.56
YLF	5.30 ± 2.70	16.69 ± 2.56	4.38 ± 3.93	13.58 ± 0.58
Donepezil	95.68 ± 0.46	101.34 ± 0.71	98.10 ± 0.27	98.86 ± 0.36

Abbreviations: DLB, Branch of Dal Ligarba; DLF, Fruit of Dal Ligarba; DLL, Leaf of Dal Ligarba; YLB, Branch of Yer Ligarba; YLF, Fruit of Yer Ligarba; YLL, Leaf of Yer Ligarba.

In the butyrylcholinesterase enzyme inhibition assay, donepezil exhibited inhibition of 98.10% and 98.86% at concentrations of 500 and 1000 μg/mL, respectively. Among the extracts, DLF exhibited the highest activity but could not reach the effect of donepezil, showing inhibition of 11.91% and 16.37% at the same concentrations. This was followed by YLB, DLB, YLF, and YLL. The lowest activity was observed in DLL.

### 3.8 Quantification by LC–MS/MS Method

**FIGURE 2:**
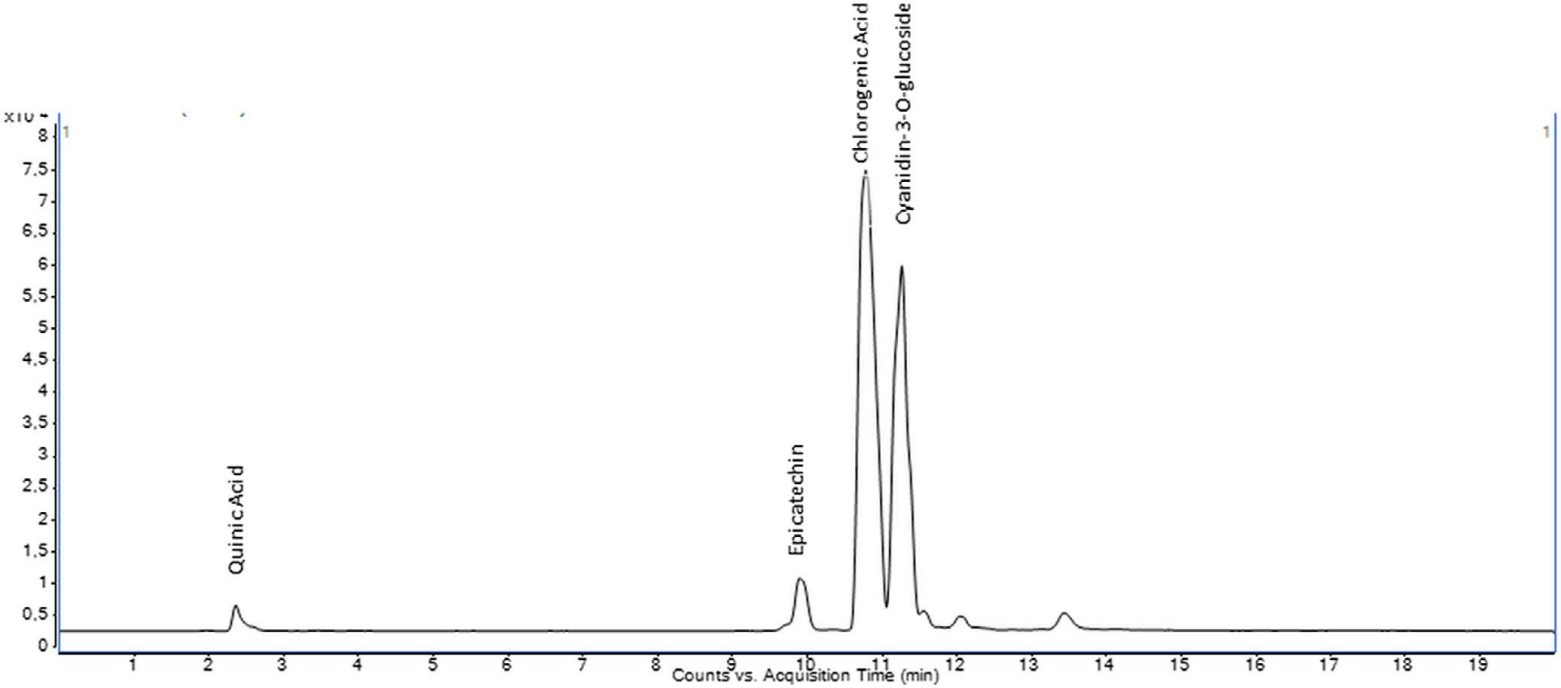
Chromatogram of phenolic compounds in the branch extract of Dal Ligarba (DLB).

**FIGURE 3:**
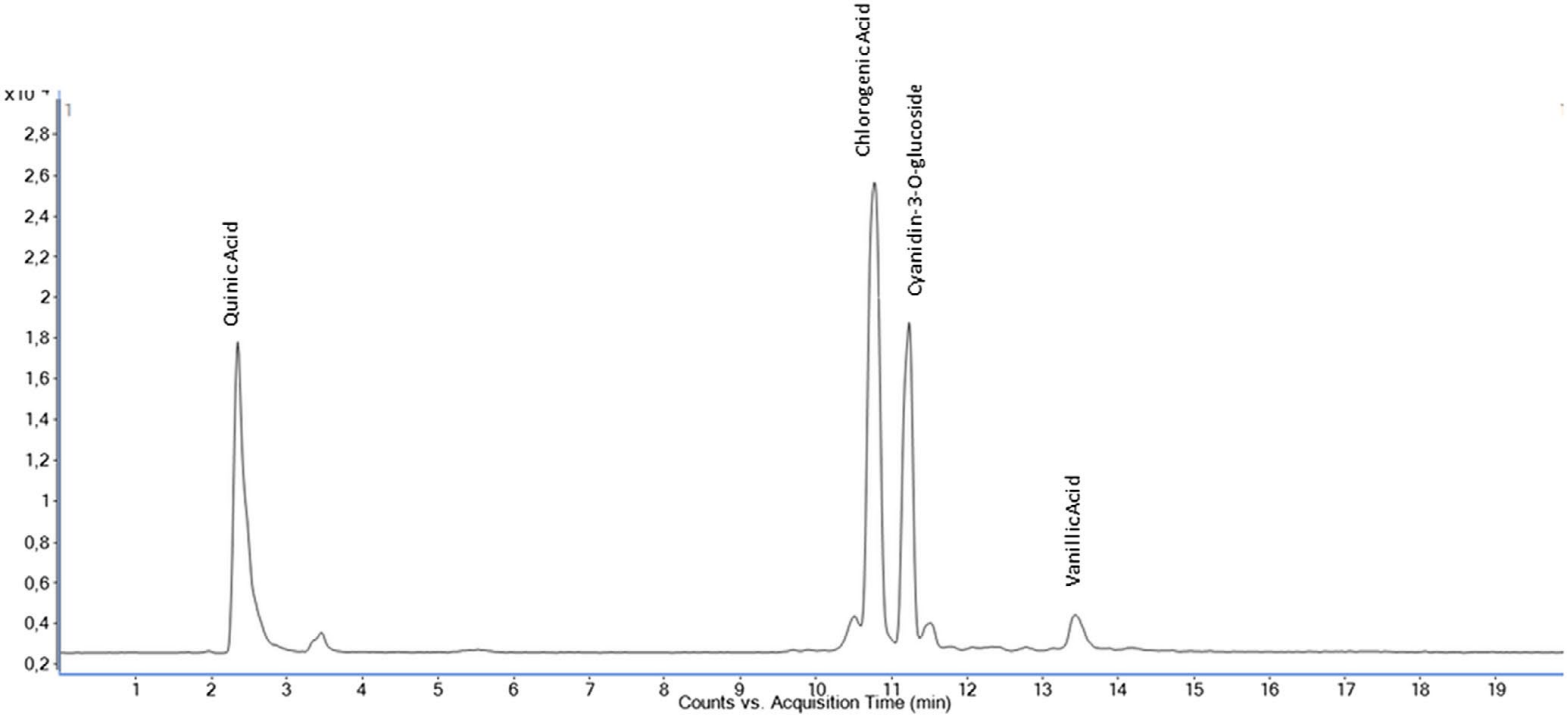
Chromatogram of phenolic compounds in the fruit extract of Dal Ligarba (DLF).

**FIGURE 4:**
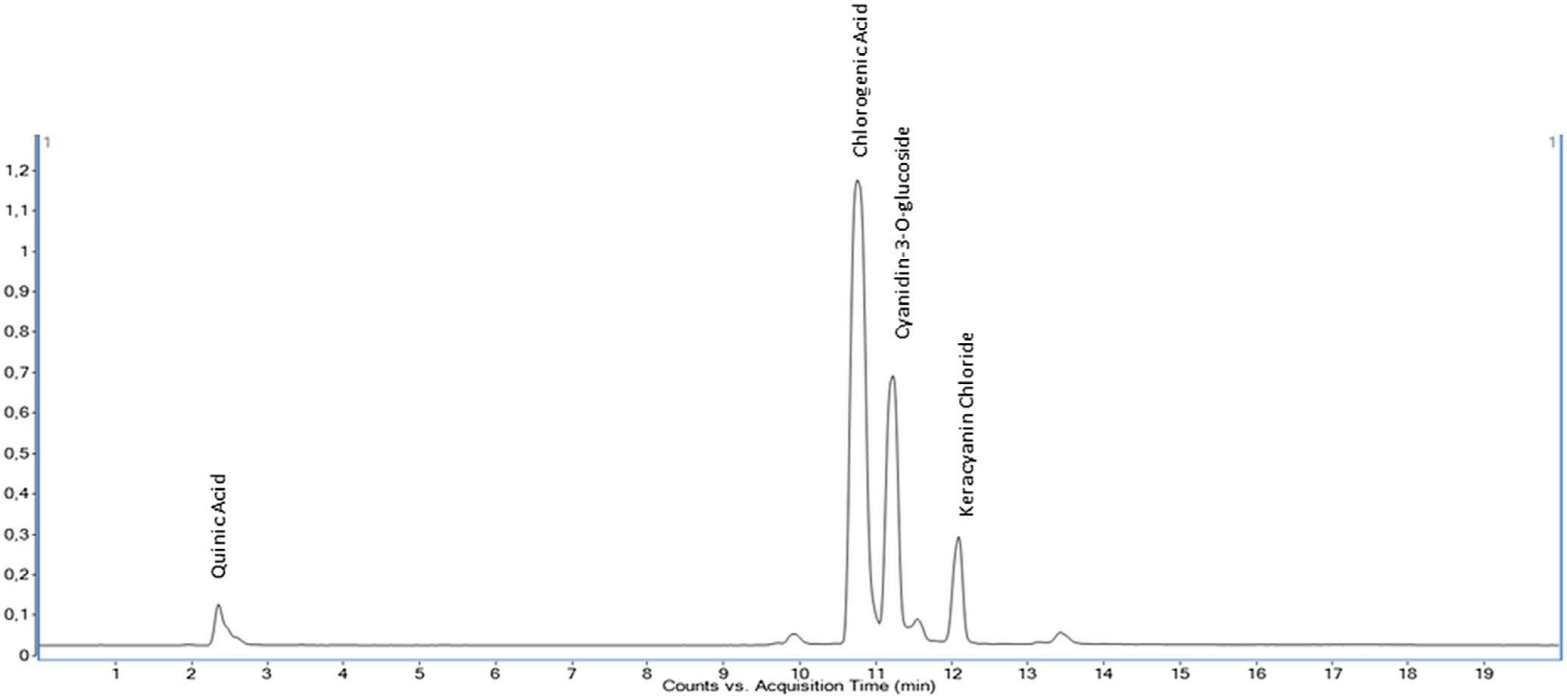
Chromatogram of phenolic compounds in the leaf extract of Dal Ligarba (DLL).

**FIGURE 5:**
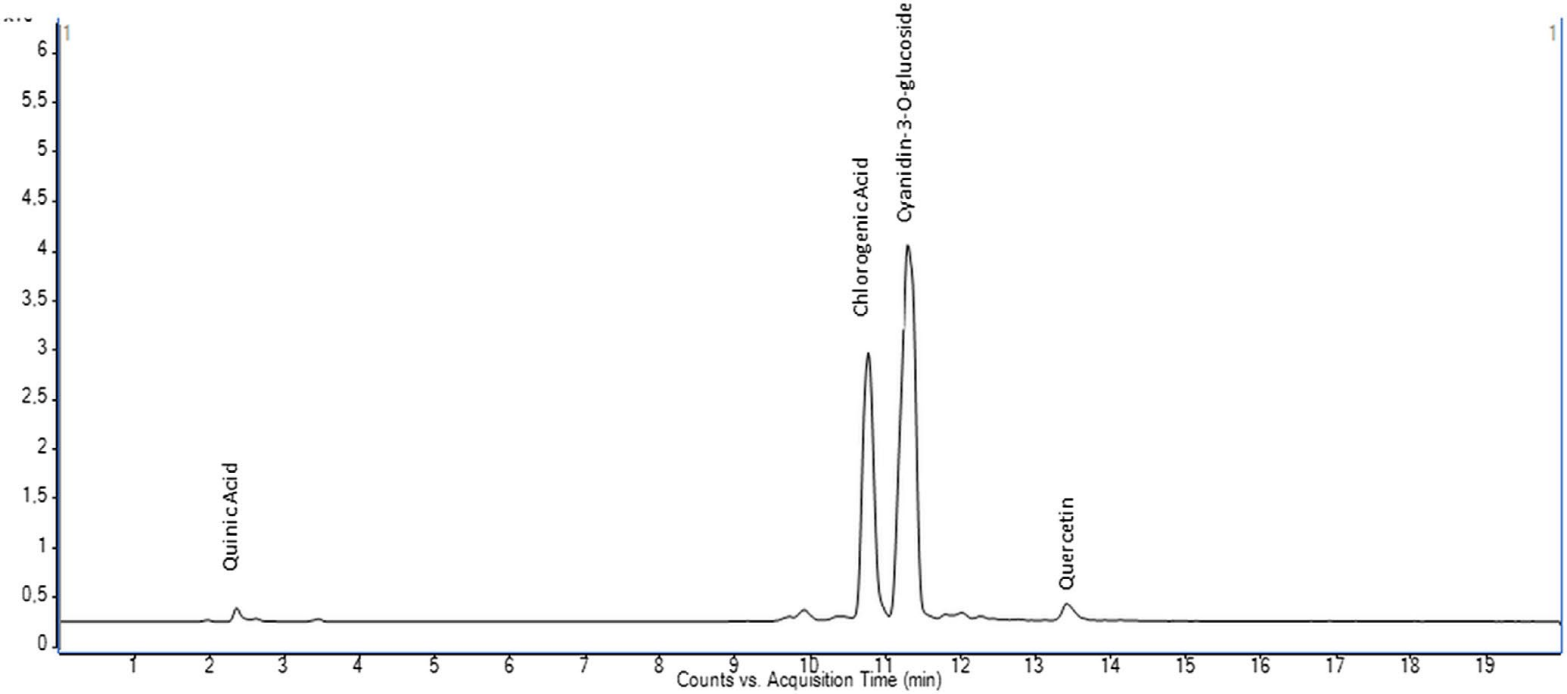
Chromatogram of phenolic compounds in the branch extract of Yer Ligarba (YLB).

**FIGURE 6:**
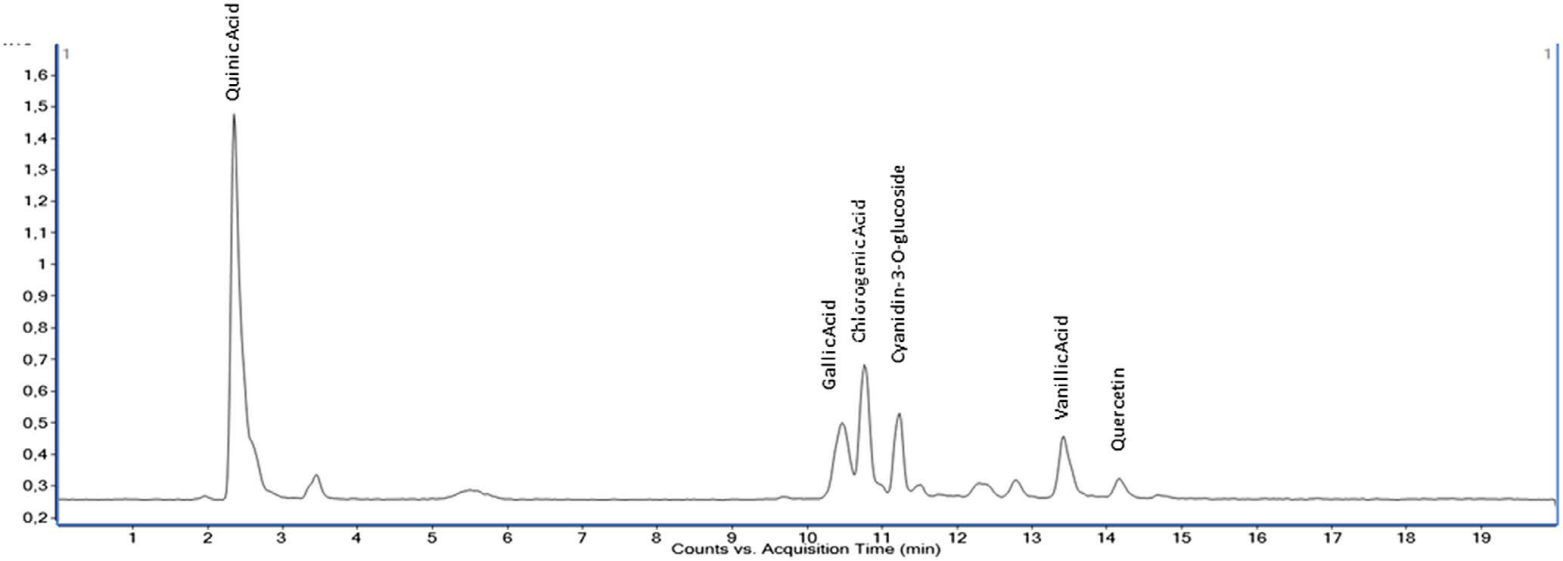
Chromatogram of phenolic compounds in the fruit extract of Yer Ligarba (YLF).

**FIGURE 7:**
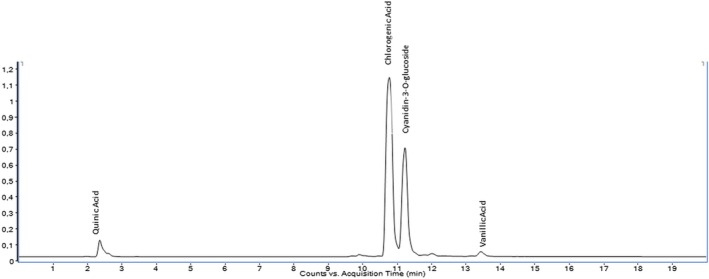
Chromatogram of phenolic compounds in the leaf extract of Yer Ligarba (YLL).

The quantitative analysis of 35 phenolic compounds in methanolic extracts, conducted using LC–MS/MS, identified 20 compounds across the samples, as detailed in Table 10. Chlorogenic acid was the most abundant phenolic compound, particularly in the DLL and YLL samples, with concentrations of 58,569.80 and 55,630.25 ng/mL, respectively. Notably, quinic acid also exhibited high levels, with concentrations of 51,325.63, 33,158.12, and 31,444.77 ng/mL in the DLF, DLL, and YLF samples, respectively. Furthermore, significant quantities of cyanidin‐3‐O‐glucoside (17,401.12 ng/mL in DLL) and catechin (10,431.49 ng/mL) were detected, highlighting the diverse phenolic profile of the extracts. The chromatograms of phenolic compounds in the extracts are presented in Figures 2, 3, 4, 5, 6, 7.

**TABLE 10:** Quantitative analysis of different phenolic compounds via LC–MS/MS.

No	Compound	Samples	Final concentration (ng/mL)
1.	Quinic acid	DLB	11,613.48
		DLF	51,325.63
		DLL	33,158.12
		YLF	31,444.77
		YLL	28,793.17
		YLB	3055.59
2.	Fumaric acid	DLB	4.90
		DLF	ND
		DLL	ND
		YLF	0.08
		YLL	ND
		YLB	0.50
3.	Gallic acid	DLB	ND
		DLF	52.19
		DLL	ND
		YLF	139.02
		YLL	ND
		YLB	ND
4.	Keracyanin chloride	DLB	15.40
		DLF	10.57
		DLL	1157.15
		YLF	8.79
		YLL	15.92
		YLB	100.62
5.	Cyanidin‐3‐O‐glucoside	DLB	1277.14
		DLF	1324.96
		DLL	17,401.12
		YLF	2353.10
		YLL	859.84
		YLB	431.39
6.	Chlorogenic acid	DLB	35,394.37
		DLF	9324.18
		DLL	58,569.80
		YLF	1448.40
		YLL	55,630.25
		YLB	10,357.43
7.	Catechin	DLB	7013.19
		DLF	ND
		DLL	219.61
		YLF	ND
		YLL	1461.32
		YLB	10,431.49
8.	Peonidin‐3‐O‐glucoside	DLB	5.59
		DLF	487.42
		DLL	1.34
		YLF	849.09
		YLL	3.10
		YLB	12.67
9.	Epicatechin	DLB	6186.67
		DLF	ND
		DLL	76.16
		YLF	ND
		YLL	1221.19
		YLB	9191.46
10.	Caffeic acid	DLB	ND
		DLF	126.72
		DLL	369.96
		YLF	ND
		YLL	52.66
		YLB	ND
11.	Vanillic acid	DLB	112.11
		DLF	1710.25
		DLL	45.74
		YLF	5634.69
		YLL	65.26
		YLB	ND
12.	Syringic acid	DLB	ND
		DLF	48.31
		DLL	ND
		YLF	46.93
		YLL	ND
		YLB	ND
13.	Naringin	DLB	ND
		DLF	ND
		DLL	ND
		YLF	ND
		YLL	ND
		YLB	15.76
14.	Ellagic acid	DLB	ND
		DLF	ND
		DLL	ND
		YLF	ND
		YLL	623.24
		YLB	ND
15.	Hesperidin	DLB	1537.93
		DLF	44.10
		DLL	2570.40
		YLF	ND
		YLL	54.54
		YLB	81.09
16.	p‐Coumaric acid	DLB	13.15
		DLF	ND
		DLL	ND
		YLF	37.91
		YLL	ND
		YLB	ND
17.	Ferulic acid	DLB	ND
		DLF	ND
		DLL	1226.98
		YLF	ND
		YLL	943.05
		YLB	ND
18.	Rosmarinic Acid	DLB	15.30
		DLF	12.98
		DLL	12.47
		YLF	43.79
		YLL	43.12
		YLB	46.17
19.	Resveratrol	DLB	2.06
		DLF	2.58
		DLL	2.18
		YLF	2.72
		YLL	2.08
		YLB	2.41
20.	Quercetin	DLB	624.43
		DLF	351.06
		DLL	602.42
		YLF	320.54
		YLL	574.90
		YLB	289.19

Abbreviations: DLB, Branch of Dal Ligarba; DLF, Fruit of Dal Ligarba; DLL, Leaf of Dal Ligarba; YLB, Branch of Yer Ligarba; YLF, Fruit of Yer Ligarba; YLL, Leaf of Yer Ligarba.

### 3.9 Relationships Among Phytochemical Parameters and Multivariate Pattern Analysis

**FIGURE 8:**
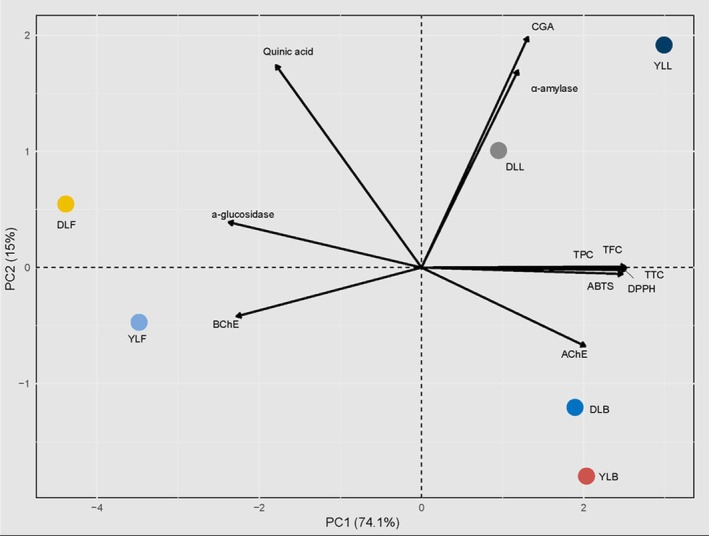
PCA biplot illustrating sample clustering based on phytochemical and enzymatic profiles. TPC refers to total phenolic content, TFC to total flavonoid content, and TTC to total tannin content. DPPH^•+^ and ABTS^•+^ represent radical scavenging activities assessed by DPPH (2,2‐diphenyl‐1‐picrylhydrazyl) and ABTS (2,2′‐azino‐bis(3‐ethylbenzothiazoline‐6‐sulfonic acid)) assays, respectively. α‐Glucosidase and α‐amylase indicate enzyme inhibition activities related to carbohydrate metabolism. AChE and BChE refer to acetylcholinesterase and butyrylcholinesterase inhibition activities, respectively. CGA denotes chlorogenic acid, while Quinic A. represents quinic acid.

Principal component analysis (PCA) revealed that the first principal component (PC1) accounted for 74.1% of the total variance, while the second component (PC2) explained an additional 15%. High positive loadings for TPC, TFC, TTC, ABTS, and DPPH were observed along PC1, indicating strong interrelationships among these antioxidant‐related variables. CGA and α‐amylase also contributed substantially to the variability, displaying significant loadings on both PC1 and PC2. The sample YLL exhibited distinct clustering, characterized by elevated TPC, TFC, and antioxidant capacity, whereas DLF and YLF clustered separately, associated with lower antioxidant potential and differing enzymatic activities. Overall, the PCA effectively discriminated the samples based on their phytochemical profiles and enzymatic characteristics (Figure 8).

**FIGURE 9:**
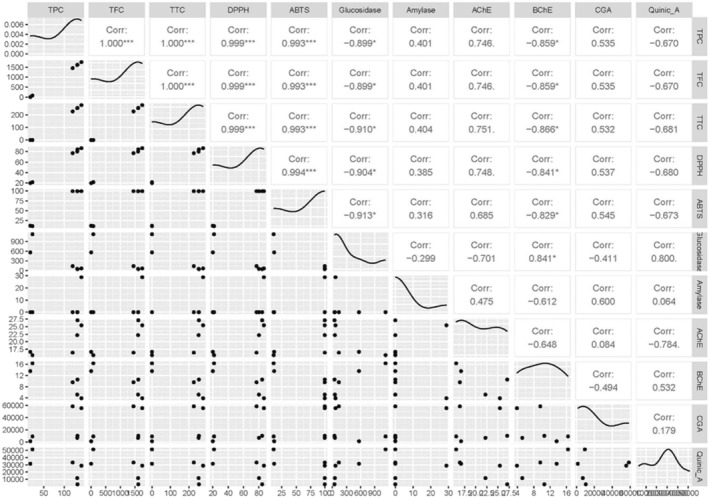
Correlation matrix among phytochemical contents, antioxidant activities, and enzyme inhibition parameters. TPC refers to total phenolic content, TFC to total flavonoid content, and TTC to total tannin content. DPPH^•^ and ABTS^•+^ represent radical scavenging activities assessed by DPPH (2,2‐diphenyl‐1‐picrylhydrazyl) and ABTS (2,2′‐azino‐bis(3‐ethylbenzothiazoline‐6‐sulfonic acid)) assays, respectively. α‐Glucosidase and α‐amylase indicate enzyme inhibition activities related to carbohydrate metabolism. AChE and BChE refer to acetylcholinesterase and butyrylcholinesterase inhibition activities, respectively. CGA denotes chlorogenic acid content, while Quinic A. represents quinic acid content. DLB corresponds to the branch of Dal Ligarba, DLL to the leaf of Dal Ligarba, DLF to the fruit of Dal Ligarba, YLB to the branch of Yer Ligarba, YLL to the leaf of Yer Ligarba, and YLF to the fruit of Yer Ligarba.

According to the results of the correlation analysis, very strong and positive relationships were observed among TPC, TFC, TTC, DPPH, and ABTS variables (*r* > 0.99, *p* < 0.001). This indicates that total phenolic and flavonoid contents are highly associated with antioxidant capacities. Strong and negative correlations were found between glucosidase activity and TPC, TFC, TTC, and DPPH (*r* ≈ −0.90, *p* < 0.05), suggesting that phenolic compounds may play a role in enzyme inhibition. Positive correlations were detected between AChE activity and phenolic contents, whereas BChE showed negative correlations. Compounds such as CGA and quinic acid exhibited moderate relationships with the studied variables. Overall, the results demonstrate that phytochemical contents significantly influence antioxidant activities and enzymatic inhibition parameters (Figure 9).

## 4 Discussion

### 4.1 Antioxidant Activity

*V. myrtillus* is among the most recognized and consumed fruits due to its important role in protecting against diseases. The known health benefits of bilberries have been attributed to different phenolic compounds with various health‐protective effects. Fruits are a source of various micronutrients with important health benefits such as phenolic compounds, vitamins, and sugars (Ciulca et al. 2021). Especially ripe fruits contain high amounts of anthocyanins, which are considered to be the most important plant pigments (Faria et al. 2005). Studies show that the concentration of anthocyanins is 70 times higher in ripe fruits compared to green fruits due to changes that cause a general decrease in the synthesis of other polyphenols and an intensification of anthocyanin synthesis during bilberry ripening (Gibson et al. 2013). Due to this rich content of blueberry extracts, it has been recorded that they have positive effects on many diseases, such as type 2 diabetes, retinal inflammation, and cancer. One of the mechanisms underlying these effects is seen as free radical scavenging effects. Antioxidants are important to prevent and protect against the effects of unstable free radicals, which damage different body biomolecules and cause many serious health problems, from cancer to heart disease. Eating antioxidant‐rich foods, especially in our daily lives, can be an effective way to reduce the likelihood of encountering these diseases (Martín‐Aragón et al. 1998).

When the studies are examined, a correlation is observed between the total phenolic compound content and antioxidant capacity (Babbar et al. 2011). Our study also gave results in this direction. It is possible to say that the extract with the highest total phenolic content has a stronger antioxidant capacity. Although studies generally focus on fruits, our study revealed that leaves and branches also have high antioxidant capacity.

### 4.2 *α*‐Glucosidase and *α*‐Amylase Inhibitory Activities

*V. myrtillus* (bilberry) leaf is traditionally used in southeastern Europe for diabetes management. A study investigated the potential of bilberry leaf extracts to inhibit carbohydrate‐hydrolyzing enzymes. The hydroethanolic extract (ethanol/H_2_O, 8:2, v/v) demonstrated significant *α*‐glucosidase inhibitory activity, with an IC_50_ value of 0.29 ± 0.02 mg/mL. This effect was statistically comparable to the standard antidiabetic drug acarbose, which showed an IC_50_ of 0.50 ± 0.01 mg/mL (Bljajić et al. 2017).

Another research evaluated the *α*‐amylase and *α*‐glucosidase inhibitory activities of methanol, ethanol, acetone, and water extracts of *Vaccinium myrtillus* fruit (VMF) at concentrations of 10–250 μg/mL. The methanol extract exhibited the most potent *α*‐amylase inhibitory activity, with an IC_50_ value of 61.38 ± 1.40 μg/mL, followed closely by the ethanol extract with an IC_50_ value of 65.52 ± 1.19 μg/mL. Both extracts showed moderate *α*‐glucosidase inhibitory activity. Statistical analyses revealed a significant correlation between the chemical composition and biological activity of the extracts. Anthocyanins were identified as major contributors to the *α*‐amylase inhibition activity, highlighting their role in the antidiabetic effects of VMF (Guder et al. 2015).

The study aimed to evaluate the inhibitory effects of *V. myrtillus* (bilberry) extracts on the key enzyme *α*‐amylase, which is associated with type 2 diabetes. This was the first investigation of the inhibitory properties of wild‐growing bilberries from Bulgaria against carbohydrate‐hydrolyzing enzymes. Bilberry extracts were analyzed for α‐amylase inhibition. HPLC analysis identified the main flavonols, anthocyanins, and stilbenes in the extracts. The predominant flavonols were quercetin derivatives, notably quercetin rutinoside, while the key anthocyanins were delphinidin‐3‐galactoside and malvidin‐3‐glucoside in both aqueous and organic extracts. The extracts demonstrated strong *α*‐amylase inhibitory activity, with IC_50_ values ranging from 20.8 to 194.8 μg GAE/mL (Karcheva‐Bahchevanska et al. 2023).

Another study compared the *α*‐amylase inhibitory activities of ethanol and freeze‐dried extracts prepared from the leaves and aerial parts of *V. myrtillus* (VM) and *V. vitis‐idaea* (VV) collected in Latvia. Among the extracts, VM samples demonstrated stronger *α*‐amylase inhibitory activity compared to VV samples. Additionally, freeze‐dried extracts exhibited higher inhibitory activity than ethanol extracts (Cvetkova et al. 2024).

The present study's findings demonstrate that the branches and leaves of *Dal Ligarba* (DLB and YLB) exhibit superior α‐glucosidase inhibition compared to acarbose, with IC_50_ values of 53.73 and 44.60 μg/mL, respectively. These results surpass the α‐glucosidase inhibition capacity of hydroethanolic extracts of *Vaccinium myrtillus* leaves, which showed an IC_50_ value of 0.29 mg/mL (290 μg/mL), comparable to acarbose's IC_50_ of 0.50 mg/mL in a prior study by Bljajić et al. Additionally, while the α‐glucosidase inhibitory activity of *V. myrtillus* fruits reported by Guder et al. (2015) was moderate, the inhibitory potential of DLB and YLB in this study was significantly higher.

In terms of α‐amylase inhibition, the current study found YLL to be the only effective extract, with 28.77% inhibition at 5 mg/mL, compared to acarbose's 65.66% inhibition. This effect aligns with the relatively lower α‐amylase inhibition reported for *Vaccinium myrtillus* extracts in previous research, with IC_50_ values ranging from 20.8 to 194.8 μg GAE/mL (Karcheva‐Bahchevanska et al. 2023) and freeze‐dried extracts of *Vaccinium myrtillus* leaves showing stronger inhibition compared to ethanol extracts (Cvetkova et al. 2024).

Overall, the branches and leaves of Dal Ligarba emerge as promising candidates for α‐glucosidase inhibition, outperforming both acarbose and several *V. myrtillus* extracts. However, their *α*‐amylase inhibitory potential remains relatively lower, indicating a more selective enzyme inhibition profile compared to *V. myrtillus* . These findings highlight the therapeutic potential of Dal Ligarba for managing type 2 diabetes, particularly through *α*‐glucosidase inhibition.

### 4.3 Anticholinesterase Inihibitory Activities

A study investigated the impact of heat treatment combined with enzyme preparations on the anticholinesterase activity of bilberry juices. The results showed that pre‐heating the bilberries at 80°C–90°C for 5 min before juice extraction significantly increased both anti‐acetylcholinesterase (AChE) and anti‐butyrylcholinesterase (BChE) activities compared to non‐heated juices (processed at 50°C–55°C for 2 h). Among the enzyme preparations used, juices produced with Panzym BE XXL demonstrated the highest enhancement in anti‐AChE activity after pre‐heating, with the increase being statistically significant (*p* < 0.05). Similarly, anti‐BChE activity also improved following heat pretreatment (Borowiec and Szwajgier 2020).

Another study screened selected fruits and vegetables for cholinesterase inhibitors (ChEIs) and found that bilberry fruit extract effectively inhibited both acetylcholinesterase (AChE) and butyrylcholinesterase (BChE) activities. Further purification and characterization of the active compounds from bilberry fruit using HPLC‐UV, FT‐IR, NMR, and LC–MS revealed that the ChEIs were derivatives of chlorogenic acid and benzoic acid (Borowiec et al. 2014).

The results of our study demonstrated that none of the tested extracts exhibited cholinesterase inhibition activity comparable to that of the standard compound, donepezil. Among the extracts, YLB showed the highest inhibition of acetylcholinesterase (AChE) activity (27.08% at 100 μg/mL), while DLF exhibited the highest butyrylcholinesterase (BChE) inhibition (16.37% at 1000 μg/mL). These findings align with previous studies, such as Borowiec and Szwajgier (2020), which highlighted the influence of pre‐heating treatments and enzymatic preparations in enhancing the anticholinesterase activity of bilberry juice. However, the activity levels in our extracts were significantly lower, possibly due to differences in plant material, extraction methods, or absence of similar heat treatment protocols. Additionally, Borowiec et al. (2014) identified chlorogenic and benzoic acid derivatives as key cholinesterase inhibitors in bilberry fruit, underscoring the potential importance of specific phenolic compounds. While our study did not reach the inhibition levels observed in these studies, it provides valuable insights into the moderate cholinesterase inhibition potential of the tested plant extracts and highlights the need for further optimization of extraction methods or pre‐treatment processes to enhance bioactivity.

### 4.4 Quantification by LC–MS/MS Method

Chlorogenic acid is a phenolic compound prevalent in plant‐based diets. Renowned for its hypoglycemic, hypolipidemic, anti‐inflammatory, and antioxidant properties, it has gained attention for its role in managing DM. It not only helps prevent DM but also mitigates complications such as diabetic nephropathy, retinopathy, and peripheral neuropathy (Yan et al. 2020). Green coffee, an unroasted bean rich in chlorogenic acid and antioxidants, was reviewed for its health benefits against diabetes, obesity, and hypertension. The impact of roasting on chlorogenic acid content, extraction methods, and its anti‐diabetic and anti‐obesity potential was evaluated (Pimpley et al. 2020). The effects of chlorogenic acid on glucose and lipid metabolism, adiponectin receptors, and signaling pathways in late‐stage diabetic db/db mice were investigated. Chlorogenic acid (80 mg/kg/day) was administered for 12 weeks. Significant reductions in body fat, fasting plasma glucose, HbA1c, and kidney fibrosis markers were observed, along with increases in adiponectin levels, AMPK phosphorylation, and PPAR‐α expression (Jin et al. 2015).

With the growing aging population, neurodegenerative diseases like AD have become more prevalent. Coffee, containing chlorogenic acids, is known for its antioxidant properties that may protect neurological health. The neuroprotective effects of chlorogenic acids have been supported by studies showing their ability to reduce oxidative stress and prevent cell apoptosis by regulating reactive oxygen species and apoptotic proteins. Given their high bioavailability, chlorogenic acids are considered to have potential in alleviating AD (Anggreani and Lee 2017). The potential benefits of chlorogenic acid for cognition and neurological health have been explored. Chlorogenic acid is associated with neuroprotection, cardioprotection, and reduced blood pressure, with evidence indicating its potential to protect against neurological degeneration caused by oxidative stress (Heitman and Ingram 2017).

The identification of chlorogenic acid as the most abundant phenolic compound in the methanolic extracts from the DLL, YLL, and DLB samples is particularly noteworthy, given the extensive body of research supporting its health‐promoting potential. The variation in chlorogenic acid concentrations across the samples highlights its widespread presence in plant‐based diets, which is consistent with its well‐documented pharmacological properties. Chlorogenic acid has been studied extensively for its diverse biological activities, including its hypoglycemic, hypolipidemic, anti‐inflammatory, and antioxidant effects, making it a key compound in managing various health conditions. Research has consistently emphasized the therapeutic significance of chlorogenic acid in the context of DM. It has been recognized for its multifaceted role in preventing DM and alleviating complications such as diabetic nephropathy, retinopathy, and peripheral neuropathy. These findings align with the high concentrations of chlorogenic acid observed in the current study, supporting the hypothesis that this compound may contribute to the prevention and management of DM and its associated complications. Its ability to modulate glucose metabolism and reduce oxidative stress positions chlorogenic acid as a promising agent in the management of metabolic disorders. Beyond its role in diabetes management, chlorogenic acid has also demonstrated neuroprotective potential. With the growing aging population and the increasing prevalence of neurodegenerative diseases, such as AD, the antioxidant properties of chlorogenic acid are gaining significant attention. Studies have highlighted its ability to reduce oxidative stress and prevent apoptosis, mechanisms that are particularly relevant in the context of neurodegenerative diseases. The neuroprotective effects of chlorogenic acid are further supported by evidence suggesting its potential to protect against oxidative stress‐induced neurological degeneration. The current study's findings, which show substantial levels of chlorogenic acid, reinforce these results, suggesting that the compound could contribute to the maintenance of neurological health, particularly in age‐related conditions like AD.

In conclusion, the identification of chlorogenic acid as a dominant phenolic compound in the methanolic extracts provides important insights into its therapeutic potential. Given its established benefits in managing diabetes, metabolic syndrome, and neurodegenerative diseases, chlorogenic acid emerges as a key bioactive compound with broad health‐promoting effects. The high concentrations detected in this study emphasize its importance as a potential therapeutic agent in plant‐based diets aimed at addressing chronic diseases. However, further research is needed to fully elucidate its mechanisms of action and to assess its clinical efficacy, paving the way for its incorporation into targeted therapeutic strategies.

## 5 Conclusion

In conclusion, this study provides a comprehensive evaluation of the phytochemical composition and biological activities of different parts of wild *V. myrtillus* (bilberry) plants. The results highlight the significant biological potential of bilberry, particularly its antioxidant, antidiabetic, and neuroprotective properties. This study is one of the most comprehensive investigations conducted on bilberry samples collected from the wild in Turkey, analyzing various parts of the plant in detail. Among the extracts, the YLL extract exhibited the highest values for total phenolic, flavonoid, and tannin contents, alongside the highest antioxidant activity, as demonstrated by its impressive DPPH radical scavenging inhibition. Notably, the DLB extract showed exceptional antidiabetic potential with 98.67% α‐glucosidase enzyme inhibition, surpassing acarbose at the same concentration. Additionally, the leaf extracts, DLL and YLL, demonstrated potent antioxidant effects, while the highest cholinesterase inhibitory activities were observed in the YLB and DLF extracts. Chlorogenic acid emerged as the most abundant phenolic compound, particularly in the DLL extract, suggesting its significant contribution to the plant's bioactivity. These findings underscore the potential of *V. myrtillus* as a valuable source of bioactive compounds with therapeutic applications, particularly in managing diabetes and neurodegenerative diseases, such as AD. Further research into the underlying mechanisms of action and clinical validation could pave the way for the incorporation of bilberry extracts into functional foods and pharmaceutical applications.

## Author Contributions

**Hafize Yuca:** conceptualization (equal), data curation (equal), formal analysis (equal), funding acquisition (equal), investigation (equal), methodology (equal), visualization (equal), writing – original draft (equal). **Beyzanur Ayar:** conceptualization (equal), data curation (equal), formal analysis (equal), resources (equal), visualization (equal), writing – original draft (equal). **Bilge Aydın:** conceptualization (equal), data curation (equal), formal analysis (equal), investigation (equal), resources (equal), visualization (equal), writing – original draft (equal). **Furkan Çoban:** conceptualization (equal), data curation (equal), formal analysis (equal), writing – original draft (equal). **Songül Karakaya:** conceptualization (equal), data curation (equal), formal analysis (equal), investigation (equal), methodology (equal), supervision (equal), validation (equal), visualization (equal), writing – original draft (equal), writing – review and editing (equal).

## Ethics Statement

The authors have nothing to report.

## Conflicts of Interest

The authors declare no conflicts of interest.

## Data Availability

The data supporting the findings of this study are available from the corresponding author upon reasonable request.
